# Comprehensive analysis of predictive factors for upstaging in intraprostatic cancer after radical prostatectomy: Different patterns of spread exist in lesions at different locations

**DOI:** 10.1002/cam4.6401

**Published:** 2023-08-03

**Authors:** Shangrong Wu, Yuchen Jiang, Zhengxin Liang, Shuaiqi Chen, Guangyu Sun, Shenfei Ma, Kaifei Chen, Ranlu Liu

**Affiliations:** ^1^ Department of Urology The Second Hospital of Tianjin Medical University Tianjin China; ^2^ Tianjin Institute of Urology Tianjin China

**Keywords:** extraprostatic extension, multiparameter magnetic resonance imaging, perineural invasion, prostate cancer, radical prostatectomy

## Abstract

**Background:**

Accurate assessment of the clinical staging is crucial for determining the need for radical prostatectomy (RP) in prostate cancer (PCa). However, the current methods for PCa staging may yield incorrect results. This study aimed to comprehensively analyze independent predictors of postoperative upstaging of intraprostatic cancer.

**Methods:**

We conducted a retrospective analysis of data from intraprostatic cancer patients who underwent radical surgery between March 2019 and December 2022. Intraprostatic cancer was defined as a lesion confined to the prostate, excluding cases where multiparameter magnetic resonance imaging (mpMRI) showed the lesion in contact with the prostatic capsule. We assessed independent predictors of extraprostatic extension (EPE) and analyzed their association with positive surgical margin (PSM) status. In addition, based on the distance of the lesion from the capsule on mpMRI, we divided the patients into non‐transition zone and transition zone groups for further analysis.

**Results:**

A total of 500 patients were included in our study. Logistic regression analysis revealed that biopsy Gleason grade group (GG) (odds ratio, OR: 1.370, 95% confidence interval, CI: 1.093–1.718) and perineural invasion (PNI) (OR: 2.746, 95% CI: 1.420–5.309) were predictive factors for postoperative EPE. Both biopsy GG and PNI were associated with lateral (GG: OR: 1.270, 95% CI: 1.074–1.501; PNI: OR: 2.733, 95% CI: 1.521–4.911) and basal (GG: OR: 1.491, 95% CI: 1.194–1.862; PNI: OR: 3.730, 95% CI: 1.929–7.214) PSM but not with apex PSM (GG: OR: 1.176, 95% CI: 0.989–1.399; PNI: OR: 1.204, 95% CI: 0.609–2.381) after RP. Finally, PNI was an independent predictor of EPE in the transition zone (OR: 11.235, 95% CI: 2.779–45.428) but not in the non‐transition zone (OR: 1.942, 95% CI: 0.920–4.098).

**Conclusion:**

PNI and higher GG may indicate upstaging of tumors in patients with intraprostatic carcinoma. These two factors are associated with PSM in locations other than the apex of the prostate. Importantly, cancer in the transition zone of the prostate is more likely to spread externally through nerve invasion than cancer in the non‐transition zone.

## 1 INTRODUCTION

The past few years have witnessed an increase in the annual incidence of prostate cancer (PCa), which has become the most common cancer among males.^1^ Currently, there are many treatment options for PCa. Radical prostatectomy (RP) is the main regimen for patients with localized PCa and favorable life expectancy.^2^, ^3^ For most PCa patients, RP offers the potential for complete tumor removal, thereby maximizing treatment outcomes. Nevertheless, the evaluation of localized staging in PCa is prone to inaccuracies, leading to the selection of inappropriate treatment options that ultimately affect the patient's prognosis.

The prostate does not possess a real capsule; rather, it is enveloped by fibrous and muscular tissues commonly called the “capsule”.^4^ On T2‐weighted of multiparameter magnetic resonance imaging (mpMRI), the capsule is visualized as a smooth, low‐signal curve surrounding the prostate gland.^5^, ^6^ If the lesion extends beyond the prostate capsule, it is termed extraprostatic extension (EPE), which refers to the pathological stage ≥pT3a.^7^, ^8^ Some studies^9^, ^10^, ^11^ have demonstrated that the presence of EPE increases the risk of positive surgical margins (PSM) after RP, especially for nerve‐sparing RP.^11^ The treatment options for organ‐confined PCa and locally advanced PCa differ significantly,^3^ influencing not only the choice of surgical options but even the willingness of patients to undergo surgery. Locally advanced PCa may indicate an increased risk grade,^3^ an increased risk of PSM, and the need for further postoperative radiotherapy or long‐term endocrine therapy. Hence, improving the accuracy of preoperative clinical staging would facilitate the selection of appropriate treatment regimens and intraoperative resection boundaries.

In clinical practice, evaluating the local staging of PCa mainly relies on pelvic mpMRI.^12^, ^13^ However, mpMRI is widely thought to yield subjective and inaccurate conclusions in evaluating the extent of lesion involvement beyond the visual capsule of the prostate.^5^, ^14^, ^15^ In some cases, patients without observed EPE on mpMRI may exhibit pathological EPE, leading to PSM after surgery.^7^ Although many studies^12^, ^16^, ^17^ have attempted to identify variables or develop nomograms to predict pathological EPE, our study cohort differs from previous studies. The concept of “intraprostatic cancer” that we present in this study deviates from the accepted definition of clinically localized PCa (≤cT2c). Specifically, we restricted our study to patients with lesions located within the prostate, with a distinct normal signal tissue spacer between the lesion and the signal of the capsule, excluding patients with clinically localized PCa whose lesions were connected with the capsule on mpMRI. Our objective was to determine factors that could predict the presence of postoperative EPE in patients with intraprostatic cancer and evaluate the impact of these factors on surgical outcomes.

## 2 METHOD

We retrospectively collected data on PCa patients who underwent RP at the Second Hospital of Tianjin Medical University between March 2019 and July 2022. The baseline clinical features of patients were based on information at the time of biopsy, including age, serum prostate‐specific antigen (PSA) before biopsy, the result of digital rectal examination (DRE), lesion location on mpMRI, the number of positive biopsy cores, the percentage of unilateral maximum positive cores, perineural invasion (PNI) in biopsy pathology reports, the method of biopsy, and Gleason grade group (GG).^18^ Abnormal DRE findings were defined as the presence of palpable nodules in the prostate or hardening of the prostate. EPE was considered present if the postoperative pathology report indicated that PCa had breached the capsule or invaded the seminal vesicles, adjacent tissues, or organs (≥pT3a). The Institutional Review Committee and the Medical Ethics Committee of the Second Hospital of Tianjin Medical University approved the research protocol of this study. Informed consent was obtained from all participating patients.

Our inclusion criteria for patients were as follows: (1) confirmed pathological diagnosis of PCa by transrectal or transperineal prostate systematic with or without targeted biopsy; (2) pelvic mpMRI was performed within 3 months before biopsy; (3) absence of systemic bone metastasis by bone scan; (4) patients that underwent RP with detailed postoperative pathological information available (5) if the duration between biopsy and RP was longer than 3 months, a repeat mpMRI was conducted within 3 months before RP. (6) mpMRI revealing a distinct normal signal boundary between the lesion or abnormal signal and the capsule.

Exclusion criteria were as follows: (1) a history of androgen deprivation therapy, chemotherapy, or radiotherapy before surgery; (2) mpMRI indicating a bulge capsule^5^; (3) the loss of the space between the prostate and the rectum on mpMRI; (4) presence of pelvic lymph node enlargement on mpMRI.

MRI scans were reviewed by two experienced radiologists, and in cases of disagreement, another radiologist reviewed the mpMRI. Previously, the central zone and the transition zone were collectively referred to as the “central gland.” However, these terminologies are no longer recommended, as mpMRI can distinguish between these zones.^6^ To investigate whether the risk of EPE is higher with greater distance of the lesion from the center of the gland, we further assessed the patients by dividing them into transition zone and non‐transition zone groups based on the location of the lesion in the prostate on mpMRI. The transition zone surrounds the urethra, proximal to the verumontanum. These regions are relatively distant from the capsule. On the other hand, the non‐transition zone includes the peripheral zone, central zone, and anterior fibromuscular stroma, which surround the transition zone and are located closer to the capsule.^19^ The biopsy procedure is done under the guidance of MRI‐ultrasound fusion imaging. The systematic biopsy procedure ensures that samples are taken uniformly from various areas of the entire prostate gland. On the other hand, the targeted biopsy is specifically conducted at the location of any abnormal signal observed on the mpMRI image. Unilateral maximum positive cores percentage was calculated by the formula: the number of unilateral maximum positive cores/the number of ipsilateral biopsy cores. In addition, information on RP was also collected, including the method of surgery, and prostate volume (PV), prostate transverse diameter, prostate longitudinal diameter. The diameter of the prostate was measured by preoperative mpMRI and PV was calculated by multiplying the product of diameters in three directions by 0.52.^6^

SPSS 25.0 statistical software was used to process the data. The chi‐squared and Mann–Whitney *U*‐tests were used for comparison between the EPE and the non‐EPE groups. Logistic regression analysis was used to evaluate the association between variables and EPE. Variables with statistical significance during univariate analysis were included in the multivariate logistic analysis. A *p* < 0.05 was statistically significant. The receiver operator characteristic (ROC) curve analysis and area under the curve (AUC)^20^ were used to evaluate the predictive ability of independent factors.

## 3 RESULTS

Our study included a total of 500 PCa patients. According to the postoperative pathological report, 101 (20.2%) patients had EPE, while 399 (79.8%) patients did not have EPE. The median age of the patients was 68 years, and the remaining baseline characteristics are detailed in Table 1.

**TABLE 1:** In our study, we enrolled a total of 500 patients who underwent RP. These patients were further divided into two groups based on the presence or absence of EPE.

Variables/value	Overall (*n* = 500, 100%)	Non‐EPE (*n* = 399, 79.8%)	EPE (*n* = 101, 20.2%)	p
Age (years), Median (IQR)	68 (64–72)	68 (63–72)	69 (65–73)	0.347
PSA (continuous) (ng/mL), Median (IQR)	14.20 (8.60–24.75)	13.00 (8.11–22.00)	20.29 (12.59–38.98)	<0.001
PSA (grade) (ng/mL), *n* (%)				<0.001
<10	161 (32.2%)	145 (36.3%)	16 (15.8%)	
10–20	177 (35.4%)	143 (35.8%)	34 (33.7%)	
≥20	162 (32.4%)	111 (27.8%)	51 (50.5%)	
Result of DRE, *n* (%)				<0.001
Abnormal	192 (38.4%)	126 (31.6%)	66 (65.3%)	
Normal	308 (61.6%)	273 (68.4%)	35 (34.7%)	
Lesion location, *n* (%)				<0.001
Non‐transition zone	281 (56.2%)	204 (51.1%)	77 (76.2%)	
Transition zone	219 (43.8%)	195 (48.9%)	24 (23.8%)	
PV (cm^3^), median (IQR)	31.53 (21.10–44.64)	31.80 (21.10–45.17)	31.20 (23.95–41.45)	0.745
Prostate transverse diameter (cm), median (IQR)	4.8 (4.2–5.3)	4.8 (4.2–5.3)	4.8 (4.3–5.1)	0.859
Prostate longitudinal diameter (cm), median (IQR)	3.9 (3.0–4.5)	3.8 (3.0–4.6)	3.9 (3.0–4.5)	0.775
Rate of prostate transverse diameter/longitudinal diameter	1.27 (1.13–1.43)	1.26 (1.14–1.42)	1.27 (1.13–1.4)	0.881
Biopsy GG, *n* (%)				<0.001
1	122 (24.4%)	115 (28.8%)	7 (6.9%)	
2	115 (23.0%)	99 (24.8%)	16 (15.8%)	
3	81 (16.2%)	62 (15.5%)	19 (18.9%)	
4	148 (29.6%)	105 (26.4%)	43 (42.6%)	
5	34 (6.8%)	18 (4.5%)	16 (15.8%)	
Time from imaging to biopsy (days), median (IQR)	6 (4–7)	6 (4–7)	6 (5–7)	0.315
Time from imaging to RP (days), median (IQR)	38 (35–51)	38 (35–51)	39 (36–51)	0.123
Time from biopsy to RP (days), median (IQR)	32 (29–46)	32 (29–46)	32 (29–46)	0.278
Surgery procedure, *n* (%)				0.149
RARP	154 (30.8%)	129 (32.3%)	25 (24.8%)	
LRP	346 (69.2%)	270 (67.7%)	76 (75.2%)	
Nerve‐sparing surgery, *n* (%)				0.057
Yes	224 (44.8%)	170 (42.6%)	54 (53.5%)	
No	276 (55.2%)	229 (57.4%)	47 (46.5%)	
Biopsy procedure, *n* (%)				0.150
Transperineal	343 (68.6%)	280 (70.2%)	63 (62.4%)	
Transrectal	157 (31.4%)	119 (29.8%)	38 (37.6%)	
Total number of cores, median (IQR)	20 (15–24)	20 (15–25)	18 (15–23)	0.072
The number of positive biopsy cores, median (IQR)	5 (2–8)	4 (2–7)	7 (5–12)	<0.001
The number of unilateral maximum positive cores, median (IQR)	4 (2–6)	3 (2–5)	6 (4–8)	<0.001
The percentage of unilateral maximum positive cores, median (IQR)	37.50 (20.00–63.64)	33.33 (18.18–54.54)	65.2 (40.83–90.27)	<0.001
PNI on biopsy, *n* (%)	56 (11.2%)	29 (7.3%)	27 (26.7%)	<0.001

Abbreviations: DRE, digital rectal examination; EPE, extraprostatic extension; GG, grade group; IQR, interquartile range; LRP, laparoscopic radical prostatectomy; MRI, magnetic resonance imaging; PNI, perineural invasion; PSA, prostate‐specific antigen; PV, prostate volume; RARP, robot‐assisted radical prostatectomy; RP, radical prostatectomy.

### 3.1 The relationship between EPE and PSM

We confirmed the positive correlation between EPE and PSM, highlighting the significance of preoperative identification of EPE. The presence of PSM was determined by postoperative pathology. As shown in Table 2, the PSM rates at different locations in patients within the EPE group (apex: 28.7%; lateral: 42.6%; base: 30.7%)were significantly higher compared to the non‐EPE group (apex: 16.3%; lateral: 16%; base: 7%).

**TABLE 2:** We compared the rates of PSM between the EPE and non‐EPE groups in our study.

Variables/value	Non‐EPE (*n* = 399, 79.8%)	EPE (*n* = 101, 20.2%)	*p*
PSM at lateral of prostate, *n* (%)	64 (16.0%)	43 (42.6%)	<0.001
PSM at apex of prostate, *n* (%)	65 (16.3%)	29 (28.7%)	0.006
PSM at base of prostate, *n* (%)	28 (7.0%)	31 (30.7%)	<0.001

Abbreviations: EPE, extraprostatic extension; PCa, prostate cancer; PSM, positive surgery margin.

### 3.2 The independent predictive factors of postoperative EPE in all patients

**FIGURE 1:**
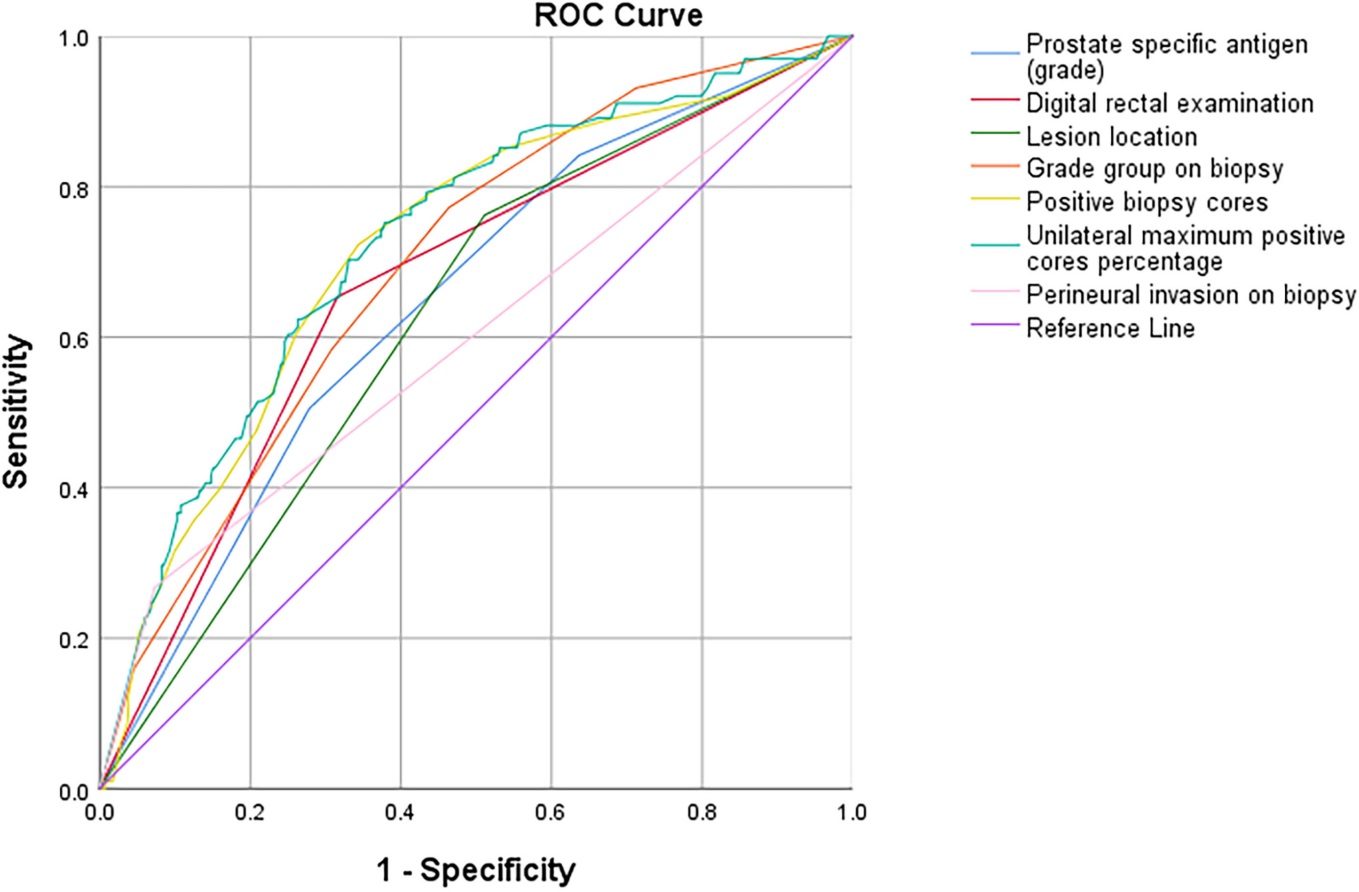
The receiver operating characteristic (ROC) curve demonstrated the efficacy of all significant factors identified in the univariate regression analysis for predicting postoperative extraprostatic extension.

Logistic regression analysis was performed on data from 500 patients (Table 3). During univariate analysis, variables that exhibited statistical significance were PSA (grade) (odds ratio, OR: 2.022, 95% confidence interval, 95% CI: 1.507–2.713, *p* < 0.001), DRE abnormalities (OR: 4.086, 95% CI: 2.577–6.479, *p* < 0.001), lesion location (OR: 3.067, 95% CI: 1.863–5.049, *p* < 0.001), GG, the number of positive biopsy cores (OR: 1.159, 95% CI: 1.104–1.217, *p* < 0.001), the percentage of unilateral maximum positive cores (OR: 1.028, 95% CI: 1.020–1.037, *p* < 0.001), and PNI (OR: 4.655, 95% CI: 2.605–8.319, *p* < 0.001). These variables were incorporated during multivariate logistic regression analysis. We found that increased GG (OR: 1.370, 95% CI: 1.093–1.718, *p* = 0.006) and PNI (OR: 2.746, 95% CI: 1.420–5.309, *p* < 0.001) were independent predictive factors of EPE. A ROC analysis was conducted for the significant factors mentioned above. The results showed the efficacy of the biopsy GG (AUC: 0.696, 95% CI: 0.642–0.751, *p* < 0.001) and PNI (AUC: 0.597, 95% CI: 0.531–0.664, *p* = 0.002) as independent factors in predicting postoperative EPE (Figure 1, Table S1).

**TABLE 3:** In our study, we performed logistic regression analysis on data from 500 patients with PCa to identify the independent factors influencing EPE.

Variables	Univariate analysis			Multivariate analysis		
	OR	95% CI	*p*	OR	95% CI	*p*
Age	1.018	0.983–1.053	0.314			
PSA (grade variable)	2.022	1.507–2.713	<0.001	1.313	0.942–1.830	0.109
Abnormal result of DRE	4.086	2.577–6.479	<0.001	1.656	0.912–3.005	0.097
Lesion location			<0.001			0.165
Transition zone (reference)	–	–		–	–	
Non‐transition zone	3.067	1.863–5.049		1.489	0.849–2.613	
PV	0.996	0.984–1.008	0.525			
Prostate transverse diameter	0.940	0.722–1.225	0.647			
Prostate longitudinal diameter	0.952	0.751–1.208	0.687			
Rate of prostate transverse diameter/ longitudinal diameter	0.925	0.354–2.569	0.925			
Biopsy GG	1.798	1.486–2.176	<0.001	1.370	1.093–1.718	0.006
Time from imaging to biopsy	1.075	0.945–1.223	0.271			
Time from imaging to RP	1.021	0.995–1.047	0.110			
Time from biopsy to RP	1.018	0.992–1.044	0.171			
Positive biopsy cores	1.159	1.104–1.217	<0.001	0.990	0.913–1.072	0.799
Unilateral maximum positive cores percentage	1.028	1.020–1.037	<0.001	1.012	0.999–1.026	0.076
PNI on biopsy	4.655	2.605–8.319	<0.001	2.746	1.420–5.309	<0.001

Abbreviations: CI, confidence interval; DRE, digital rectal examination; EPE, extraprostatic extension; GG, grade group; MRI, magnetic resonance imaging; OR, odds ratio; PCa, prostate cancer; PNI, perineural invasion; PSA, prostate‐specific antigen; PV, prostate volume.

### 3.3 The relationship between the predictive factors and the PSM

To assess the impact of the independent predictive factor of EPE on surgical outcomes, logistic regression analysis was utilized to investigate the association between these factors and PSM at different locations (Table 4). The results showed that increased GG was associated with increased PSM rates at the base (OR: 1.491, 95% CI: 1.194–1.862, *p* < 0.001) and lateral regions (OR: 1.27, 95% CI: 1.074–1.501, *p* = 0.005) of the prostate. The presence of PNI also suggested a correlation with increased risk of PSM at the base (OR: 3.730, 95% CI: 1.929–7.214, *p* < 0.001) and lateral (OR: 2.733, 95% CI: 1.521–4.911, *p* = 0.001) region of the prostate. However, GG (OR: 1.176, 95% CI: 0.989–1.399, *p* = 0.067), and PNI (OR: 1.204, 95% CI: 0.609–2.381, *p* = 0.594) were not associated with PSM at the prostate apex.

**TABLE 4:** In order to assess the impact of the aforementioned factors on surgical outcomes, we utilized logistic regression analysis to examine the association between GG and PNI variables with PSM at various locations.

Location of PSM	Apex			Base			Lateral		
Variables	OR	95% CI	*p*	OR	95% CI	*p*	OR	95% CI	*p*
Biopsy GG	1.176	0.989–1.399	0.067	1.491	1.194–1.862	<0.001	1.270	1.074–1.501	0.005
PNI	1.204	0.609–2.381	0.594	3.730	1.929–7.214	<0.001	2.733	1.521–4.911	0.001

Abbreviations: CI, confidence interval; GG, grade group; OR, odds ratio; PNI, perineural invasion; PSM, positive surgery margin.

### 3.4 The independent predictive factors of postoperative EPE in patients with lesions located in the non‐transition zone

We further analyzed 281 patients with tumors in the non‐transition zone (Table 5). After univariate logistic regression analysis, PSA (grade) (OR: 1.612, 95% CI: 1.148–2.262, *p* = 0.006), DRE abnormalities (OR: 4.216, 95% CI: 2.371–7.499, *p* < 0.001), GG (OR: 1.735, 95% CI: 1.377–2.187, *p* < 0.001), positive biopsy cores (OR: 1.142, 95% CI: 1.075–1.214, *p* < 0.001), PNI (OR: 3.102, 95% CI: 1.589–6.055, *p* = 0.001), and percentage of unilateral maximum positive cores (OR: 1.026, 95% CI: 1.016–1.037, *p* < 0.001) were included in multivariate analysis. For patients with lesions in the non‐transition zone on mpMRI, increased GG (OR: 1.383, 95% CI: 1.058–1.809, *p* = 0.018) was a significant predictive factor of EPE. However, PNI (OR: 1.942, 95% CI: 0.920–4.098, *p* = 0.082) was not associated with postoperative EPE.

**TABLE 5:** We conducted logistic regression analysis specifically for EPE using data from 281 patients with tumors located in the non‐transition zone.

Variables	Univariate analysis			Multivariate analysis		
	OR	95% CI	*p*	OR	95% CI	*p*
Age	1.040	0.999–1.084	0.056			
PSA (grade variable)	1.612	1.148–2.262	0.006	1.165	0.781–1.739	0.454
Abnormal result of DRE	4.216	2.371–7.499	<0.001	1.783	0.879–3.614	0.109
PV	1.001	0.988–1.015	0.870			
Prostate transverse diameter	1.159	0.850–1.581	0.351			
Prostate longitudinal diameter	1.132	0.854–1.501	0.389			
Rate of prostate transverse diameter/longitudinal diameter	0.665	0.193–2.293	0.519			
Time from imaging to biopsy	0.998	0.857–1.163	0.980			
Time from imaging to RP	1.023	0.992–1.055	0.144			
Time from biopsy to RP	1.023	0.992–1.054	0.146			
Biopsy GG	1.735	1.377–2.187	<0.001	1.383	1.058–1.809	0.018
Positive biopsy cores	1.142	1.075–1.214	<0.001	0.987	0.898–1.084	0.778
Unilateral maximum positive cores percentage	1.026	1.016–1.037	<0.001	1.014	0.998–1.030	0.092
PNI on biopsy	3.102	1.589–6.055	0.001	1.942	0.920–4.098	0.082

Abbreviations: CI, confidence interval; DRE, digital rectal examination; EPE, extraprostatic extension; GG, grade group; MRI, magnetic resonance imaging; OR, odds ratio; PCa, prostate cancer; PLD, prostate longitudinal diameter; PNI, perineural invasion; PSA, prostate‐specific antigen; PTD, prostate transverse diameter; PV, prostate volume; SA, surrounding area.

### 3.5 The independent predictive factors of postoperative EPE in patients with lesions located in the transition zone

About 219 patients with lesions located in the transition zone were analyzed in the present study (Table 6). Finally, only the biopsy PNI (OR: 11.235, 95% CI: 2.779–45.428, *p* = 0.001) was the independent predictive factor of EPE after multivariate logistic regression analysis.

**TABLE 6:** We conducted logistic regression analysis specifically for EPE using data from 219 patients with tumors located in the transition zone.

Variables	Univariate analysis			Multivariate analysis		
	OR	95% CI	*p*	OR	95% CI	*p*
Age	0.979	0.916–1.047	0.540			
PSA (grade variable)	2.296	1.304–4.041	0.004	1.798	0.970–3.334	0.062
Abnormal result of DRE	2.841	1.149–7.026	0.024	1.156	0.358–3.729	0.809
PV	0.977	0.949–1.006	0.114			
Prostate transverse diameter	0.610	0.349–1.066	0.083			
Prostate longitudinal diameter	0.645	0.388–1.072	0.091			
Rate of prostate transverse diameter/longitudinal diameter	2.731	0.440–16.938	0.280			
Time from imaging to biopsy	1.233	0.950–1.600	0.115			
Time from imaging to RP	0.993	0.941–1.047	0.786			
Time from biopsy to RP	0.985	0.933–1.040	0.592			
Biopsy GG	1.669	1.171–2.380	0.005	1.303	0.852–1.977	0.225
Positive biopsy cores	1.133	1.039–1.235	0.005	1.006	0.855–1.183	0.943
Unilateral maximum positive cores percentage	1.023	1.007–1.039	0.004	1.014	0.986–1.042	0.327
PNI on biopsy	8.952	2.716–29.511	<0.001	11.235	2.779–45.428	0.001

Abbreviations: CA, central area; CI, confidence interval; DRE, digital rectal examination; EPE, extraprostatic extension; GG, grade group; MRI, magnetic resonance imaging; OR, odds ratio; PCa, prostate cancer; PLD, prostate longitudinal diameter; PNI, perineural invasion; PSA, prostate‐specific antigen; PTD, prostate transverse diameter; PV, prostate volume.

### 3.6 The association of postoperative EPE with specific factors related to GG

The time from imaging to biopsy (*p* = 0.315), imaging to RP (*p* = 0.123), and biopsy to RP (*p* = 0.278) did not differ significantly between the EPE and non‐EPE groups (Table 1). As shown in Table 7, the time from imaging to biopsy was not significantly associated with EPE either in the GG <3 group (OR: 1.033, 95% CI: 0.787–1.357, *p* = 0.815) or GG ≥3 group (OR: 1.103, 95% CI: 0.951–1.279, *p* = 0.195). However, time from imaging to RP was significantly associated with EPE in the GG ≥3 group (OR: 1.033, 95% CI: 1.002–1.065, *p* = 0.037) but not in the GG <3 group (OR: 0.978, 95% CI: 0.925–1.035, *p* = 0.439). The time from biopsy to RP was not associated with EPE in the GG <3 group (OR: 0.977, 95% CI: 0.923–1.034, *p* = 0.416) and not in the GG ≥3 group (OR: 1.028, 95% CI: 0.998–1.060, *p* = 0.071).

**TABLE 7:** We employed logistic regression analysis to examine the association between specific variables and postoperative EPE in patients with different grade groups of PCa.

Variables	Grade group <3 (*n* = 237)			Grade group ≥3 (*n* = 263)		
	OR	95% CI	*p*	OR	95% CI	*p*
Time from imaging to biopsy	1.033	0.787–1.357	0.815	1.103	0.951–1.279	0.195
Time from imaging to RP	0.978	0.925–1.035	0.439	1.033	1.002–1.065	0.037
Time from biopsy to RP	0.977	0.923–1.034	0.416	1.028	0.998–1.060	0.071
PNI on biopsy	3.255	0.965–10.975	0.057	4.417	2.178–8.956	<0.001

Abbreviations: CI, confidence interval; EPE, extraprostatic extension; OR, odds ratio; PCa, prostate cancer; PNI, perineural invasion; RP, radical, prostatectomy.

In patients with GG <3, there was no significant association between PNI and EPE, but their association approached significance (OR: 3.255, 95% CI: 0.965–10.975, *p* = 0.057). However, in patients with GG ≥3, PNI was significantly associated with EPE (OR: 4.417, 95% CI: 2.178–8.956, *p* < 0.001).

## 4 DISCUSSION

After the histopathological diagnosis of PCa, determining the clinical stage of the tumor is essential to determine the choice of subsequent treatment. The classification of patients based on their specific characteristics is necessary to establish an individualized treatment plan. For patients eligible for surgery, assessing the local extent of local involvement in the prostate is most important. Standardizing the assessment of EPE can significantly improve the accuracy of clinical staging and ultimately benefit patients. Previous studies^21^, ^22^, ^23^, ^24^, ^25^ have found that the tumor volume, the length of tumor‐capsule interface, percentage of unilateral positive cores ≥33%, preoperative PSA, and Gleason score were predictive factors of postoperative EPE in localized PCa patients. However, our study further analyzed intraprostatic cancer patients and conducted a subgroup analysis based on the lesion location.

Importantly, we found that the presence of EPE in PCa patients was associated with a higher risk of PSM at all prostate locations. Kang et al.^9^ further analyzed the correlation between the position of EPE and PSM, and the results suggested that EPE in the anterior and lateral regions of the prostate was related to PSM at the basal of the prostate. In addition, Park et al.^11^ found that the nerve‐sparing RP for PCa patients with EPE could increase the risk of PSM, which suggested that the existence of EPE was related to PSM, especially for patients who received nerve‐sparing RP. The preoperative evaluation of EPE serves as a reference to assist patients' in making informed decisions regarding nerve‐sparing surgery, and if the risk of EPE is high, nerve‐sparing surgery should be discouraged because of the increased risk of PSM.^26^ In recent years, the prediction of EPE risk has been mainly performed by nomogram or based on risk factors.^22^, ^23^, ^24^, ^25^, ^27^, ^28^ However, these studies ignored the importance of imaging, or included patients with suspected EPE based on mpMRI results. During clinical practice, tumor spread is mainly assessed by imaging,^29^, ^30^ and no factor can directly reflect the local situation of the tumor as accurately as imaging. In the present study, we only included patients who demonstrated negative EPE on mpMRI, and therefore did not completely prohibiting patients from choosing a nerve‐sparing procedure.^31^ Among the included patients (Table 1), a higher percentage of patients underwent nerve‐sparing surgery in the EPE group (53.5%) than in the non‐EPE group (42.6%), and this relationship was close to being statistically significant (*p* = 0.057). We speculate this observation may be attributed to the higher GG in the EPE‐positive group and the possibility of false negatives on imaging. However, it is important to acknowledge that this retrospective study has limitations, including potential bias in patient selection. Nevertheless, the current results are not statistically different, ensuring the accuracy of the subsequent analysis.

Therefore, it is crucial to carefully assess the presence of EPE in patients undergoing surgery. Our final results showed that biopsy GG (OR: 1.370, *p* = 0.006) and PNI on biopsy (OR: 2.746, *p* < 0.001) were independent predictive factors of EPE in all intraprostatic cancer patients. We converted the Gleason score into grade variables according to ISUP.^18^ GG is widely acknowledged as a pathological parameter used to evaluate the degree of malignancy of PCa.^2^, ^18^ A higher grade is associated with a worse prognosis^18^ and more aggressive behavior.^32^ Numerous studies^21^, ^24^, ^25^, ^33^ have confirmed that a higher GG may be a predictor of EPE, which supports our conclusion. In addition, a higher percentage of patients with GG ≥3 (77.3% vs. 46.4%) and a lower percentage of patients with GG <3 (22.7% vs. 53.6%) were found in the EPE group compared to the non‐EPE group in our study (Table 1). Therefore, we sought to explore the association between EPE and GG‐related factors in different GG subgroups. Considering the time duration between imaging, biopsy and RP, PCa with higher GG is more likely to progress within the same timeframe.^18^, ^34^ We included the time between biopsy, imaging and RP as variables in our analysis. The results showed no association between EPE and the time intervals between biopsy, imaging, and RP (Table 1). However, in the subgroup of patients with GG ≥3, the time between imaging and RP was significantly associated with EPE (Table 7), suggesting that the time to tumor progression may vary considerably between different GG groups, emphasizing the need to consider the impact of time factors when selecting surgical options for patients with high GG.

PNI refers to the presence of tumor cells within the nerve or around the nerve sheath.^35^ At the base and apex of the prostate, nerve branches penetrate the capsule surrounding the prostate periphery, which may be a potential pathway for EPE.^6^ However, the association between PNI and EPE is not universally supported by all studies. Bismar et al.^36^ analyzed 215 patients undergoing RP and found that PNI was not associated with postoperative EPE. Similarly, Egan and Bostwick^37^ suggested that PNI could not predict the existence of EPE. On the other hand, some studies^38^, ^39^, ^40^ substantiated that PNI (OR: 2.657, *p* = 0.003) may be the independent predictor of postoperative EPE. The discrepancy in findings across studies may be attributed to differences in study population. Previous studies included patients with PCa at various clinical stages, and the proportions of patients included within each stage varied, leading to different conclusions. In addition, some studies^41^, ^42^, ^43^ have pointed out that the risk of PNI may vary across different Gleason scores. We thus divided the patients into two subgroups based on GG and found that PNI was significantly associated with EPE only in the group of patients with GG ≥3 but not in the group with GG <3, suggesting the association between PNI and EPE may be dependent on GG. Nevertheless, the association between PNI and EPE approached significance in the GG <3 group, indicating that the risk of PNI should not be disregarded in PCa with a low Gleason grade. Accordingly, more studies are needed to determine the significance of PNI on biopsy in PCa with low GG. Moreover, Holmes et al.^44^ observed a higher risk of PSM after nerve‐sparing RP for patients with PNI. This highlights the need for increased vigilance in clinical practice when performing nerve‐sparing RP in patients with PNI, as their tumor stage is more likely to exceed cT2c. The extent of resection should also be considered. Ficarra et al.^45^ reported that PNI is positively related to any PSM after RP. Our results suggest that PNI on biopsy and increased GG increases the risk of basal and lateral PSM but is not associated with the positive margin of the apex position. Since resection of the prostatic apex affects urethral length retention and the recovery of urination function,^46^ our findings indicate that for patients with intraprostatic cancer, the resection range of lateral and basal position should be expanded, while the apex region can be spared to improve patient quality of life.

In the context of intraprostatic cancer, the location of the lesion within the prostate, whether closer to the periphery or closer to the center may influence the predictive factors of EPE. Therefore, we further divided the patient group into non‐transition zone and transition zone groups to determine differences in predictive factors of postoperative EPE in different lesion locations. In the non‐transition zone group, an increased biopsy GG suggested a higher risk of EPE, suggesting that biopsy GG could be used as a cautionary factor in most cases before surgery. Interestingly, in the non‐transition zone group, PNI was not a significant factor (OR: 1.942, *p* = 0.082). In contrast, in the group with lesions closer to the center of the prostate and away from the capsule, specifically, the transition zone on mpMRI, PNI was the only independent factor of EPE (OR: 11.235, *p* = 0.001).The prostate can be divided into peripheral, transition, and central zones according to the origin of the tissue. These zones exhibit heterogeneity, and the incidence and prognosis of PCa vary among them.^47^ Therefore, tumors exhibit different growth patterns in different zones. It has long been suggested that lesions originating in the peripheral or central zone of the prostate differ in their spread and invasion patterns and pathways from those in the transition zone.^48^ As is known to all, tumors can spread along nerves independently of vascular and lymphatic pathways.^35^, ^49^ Villers et al.^49^ reported that internal tumors of PCa can spread directly along nerves. Building upon this understanding, our hypothesis suggests that lesions located closer to the center of the prostate, primarily within the transition zone, may exhibit extraprostatic spread primarily through nerve invasion. Conversely, lesions located further away from the center, primarily within the peripheral zone, may spread extraprostatically through direct invasion of peripheral tissues. Although many previous studies have reported PNI as a potential predictive factor for EPE, our study provided hitherto undocumented evidence of the relationship between PNI as a predictive factor for EPE and the location of the lesion within the prostate. Our findings highlight that the significance of PNI in predicting EPE is influenced by the specific location of the lesion.

The limitations of our study should be acknowledged. First, it was a single‐center study, and the accuracy of the results needs to be verified by more intraprostatic cancer cohorts. Second, being a retrospective clinical study, the inclusion of patients may introduce selection bias. Finally, the interpretation of mpMRI readings is subjective as it is performed by clinicians and radiologists, which may introduce bias into the final results.

## 5 CONCLUSION

In conclusion, our findings suggest that PNI and higher GG on biopsy in the Chinese population are independent predictive factors for postoperative EPE in patients with intraprostatic cancer determined by mpMRI. Furthermore, in PCa patients with PNI and higher GG, surgeons can consider performing a more extensive resection of the basal and lateral regions of the prostate while sparing the apical region. Finally, for patients with lesions located in the transition zone on mpMRI, the presence of PNI on biopsy may indicate potential tumor upstaging. In contrast, PNI is not a significant factor when the patient's lesion is located in the peripheral zone or central zone on mpMRI, suggesting that there are distinct patterns of cancer spread within different zones of the prostate.

## AUTHOR CONTRIBUTIONS

**Shangrong Wu:** Data curation (equal); formal analysis (equal); resources (equal); software (equal); writing – original draft (lead). **Yuchen Jiang:** Data curation (equal); writing – original draft (equal). **Zhengxin Liang:** Data curation (equal). **Shuaiqi Chen:** Data curation (equal). **Guangyu Sun:** Data curation (equal); software (equal). **Shenfei Ma:** Data curation (equal); resources (equal). **Kaifei Chen:** Conceptualization (equal); data curation (equal). **Ranlu Liu:** Data curation (equal); writing – review and editing (lead).

## FUNDING INFORMATION

Science and Technology Project of Tianjin Health Committee, Fund number: ZC20116. Tianjin Key Medical Discipline (Specialty) Construction Project, Fund number: TJYXZDXK‐023A.

## CONFLICT OF INTEREST STATEMENT

The authors declare no conflict of interest.

## ETHICS STATEMENT

The authors are accountable for all aspects of the work in ensuring that questions related to the accuracy or integrity of any part of the work are appropriately investigated and resolved. All procedures performed in studies involving human participants were in accordance with the ethical standards of the institutional and/or national research committee and with the 1964 Helsinki declaration and its later amendments or comparable ethical standards.

## INFORMED CONSENT

Informed consent was obtained from all individual participants included in the study.

## REPORTING CHECKLIST

The authors have completed the STROBE reporting checklist.

## Supporting information


Table S1.

## Data Availability

All data generated or analyzed during this study are included in this published article.
