# Supplementary material for: Comprehensive analysis of predictive factors for upstaging in intraprostatic cancer after radical prostatectomy: Different patterns of spread exist in lesions at different locations
Source: Cancer Med. 2023 Aug 3;12(17):17776–87. doi: 10.1002/cam4.6401 (PMC10524000; doi:10.1002/cam4.6401)
Supplement: Supplementary file 1 — Table S1. [file CAM4-12-17776-s001.docx]

| **Variables** | Area under the curve | 95% confidence interval | | P value |
| --- | --- | --- | --- | --- |
|  |  | lower | upper |  |
| PSA(grade) | 0.646 | 0.588 | 0.705 | 0.000006 |
| Digital rectal examination | 0.669 | 0.609 | 0.729 | 1.56E-07 |
| Leision Location | 0.626 | 0.567 | 0.684 | 0.000096 |
| Grade group on biopsy | 0.696 | 0.642 | 0.751 | 1.10E-09 |
| Positive biopsy cores | 0.718 | 0.661 | 0.775 | 1.27E-11 |
| Unilateral maximum positive cores percentage | 0.728 | 0.673 | 0.784 | 1.26E-12 |
| perineural invasion on biopsy | 0.597 | 0.531 | 0.664 | 0.002497 |

**Supplementary Table 1**: The ROC analysis demonstrated the effectiveness of all the significant factors identified in the univariate regression analysis for predicting postoperative extraprostatic extension.The table displays the area under the curve for each factor.
